# Social Mind and Long-Lasting Disease: Focus on Affective and Cognitive Theory of Mind in Multiple Sclerosis

**DOI:** 10.3389/fpsyg.2019.00218

**Published:** 2019-02-07

**Authors:** Sara Isernia, Francesca Baglio, Alessia d’Arma, Elisabetta Groppo, Antonella Marchetti, Davide Massaro

**Affiliations:** ^1^Theory of Mind Unit, Department of Psychology, Università Cattolica del Sacro Cuore, Milan, Italy; ^2^Fondazione Don Carlo Gnocchi Onlus (IRCCS), Milan, Italy

**Keywords:** multiple sclerosis, theory of mind, quality of life, social cognition, neurodegenerative diseases, cognitive functions

## Abstract

The role of social cognition, including theory of mind (ToM), in affecting quality of life (QoL) along the course of diseases has been reported. This is a considerable aspect in chronic pathologies, such as multiple sclerosis (MS), in which supporting and maintaining QoL is of crucial importance. We aimed to investigate the relation between ToM, clinical variables and neuropsychological profile in a cohort of adults with long lasting disease, such as different clinical MS phenotypes (Relapsing Remitting -RR- versus Progressive -Pr). In particular, our study focuses on (1) how (affective and cognitive) ToM impairment occurs in different phenotypes, (2) whether MS ToM impairment is secondary to or independent from cognitive deficit and (3) whether ToM deficit impacts QoL. 42 adults with MS (18 M: 24 F, 52.38 ± 10.31 mean age, 21.24 ± 10.94 mean disease duration, 26 RR and 16 Pr) and 26 matched healthy controls (HC) (7 M: 19 F, 51.35 ± 12.42 mean age) were screened with a neuropsychological and ToM battery, assessing both affective and cognitive components. We found statistically significant groups differences in cognitive but not affective ToM, with a lower performance in PrMS than those with a RRMS disease course. Also, significant predictive effects of neuropsychological tests on ToM were identified in MS group. Finally, MS people with different level of affective ToM differed significantly in QoL. ToM deficit in moderately disabled people with MS involves cognitive but not affective ToM components with implications on QoL. It also appears to be related to cognitive performance. As neurological and neurocognitive profiles influence mentalizing in MS, ToM evaluation should be considered for inclusion in clinical screening.

## Introduction

Social cognition is the life-long evolving neurocognitive capacity needed to process social information. According to the most recent version of the American Psychiatric Association’s Diagnostic and Statistical Manual for Mental Disorders, social cognition appears as one of the six core components alongside neurocognitive functions such as memory, executive function, attention, orientation and language ability. There is general consensus that preserved social cognition contributes to long-term maintenance of people’s quality of life (QoL) (Dodell-Feder et al., 2015; An and Jang, 2018). Recent studies have highlighted the role of social cognition in affecting QoL along the course of diseases (Maat et al., 2012), especially in chronic pathologies (e.g., Bodden et al., 2010a; Phillips et al., 2011) such as multiple sclerosis (MS).

Multiple sclerosis is an auto-immune mediated neurodegenerative disease that strikes people already in early adult age (Lassmann, 2018). The chronic and unpredictable feature of this pathology leads patients to a sensitive management of the disease together with high burden in various aspects of daily life (Perrin, 2013). The course of the disease could be subjected to variation over time or remain stable from the onset of the pathology: patients can experience crisis which follow fully disability recovery for all their life, it is the case of relapsing remitting MS (RRMS), or they can initiate to do not completely remit after relapses in a secondary phase of the illness, the case of secondary progressive MS, or even they can present a constant progression of the pathology from the onset of the disease, such as in primary progressive MS. Progressive phenotypes show increased pathological alterations in brain than remitting MS (Lassmann et al., 2012). In these conditions, social cognition abilities are crucial to hold relationships (Carotenuto et al., 2017) and consequently to preserve social support network (Wineman, 1990), significant resource for a patient’s QoL.

Among social cognition components, theory of mind (ToM) plays a crucial role, as the ability of individuals to think of their own and others’ mental states to understand and predict behavior (Wimmer and Perner, 1983; Tomasello, 1999; Baglio and Marchetti, 2016). Recent works support the multi-domain nature of this construct, dividing in affective and cognitive parts, the *hot* and *cold* ToM (Poletti et al., 2012; Shamay-Tsoory and Aharon-Peretz, 2007). In particular, the affective component is responsible for the understanding of others’ emotions while cognitive ToM is recruited to understand others’ cognitive states, beliefs, thoughts and intentions. The importance of studying ToM construct analyzing its different aspects is corroborated by neuropsychological and imaging data (Shamay-Tsoory et al., 2005; Kalbe et al., 2010). Specifically, evidence supports a dissociation between cognitive and affective ToM neural correlates in the medial prefrontal cortex: dorsal areas are recruited in understanding cognitive mental states while ventral regions are involved in affective state comprehension (Shamay-Tsoory and Aharon-Peretz, 2007).

Considering that ToM is defined as being composed by affective and cognitive components (Shamay-Tsoory et al., 2007), it is not fully understood whether and how such components are damaged in MS. While several contributions documented the presence of a ToM deficit in MS (Henry et al., 2009; Banati et al., 2010; Henry et al., 2011), only two works consider ToM adopting latest multi-domain models, by focusing on both hot and cold components in MS (see for example, Roca et al., 2014; Raimo et al., 2017) reporting incongruent results. In one case it seems that both cognitive and affective components are impaired (Raimo et al., 2017) whereas in the other study only the cognitive domain appeared affected (Roca et al., 2014). Also, additional evidence documented a decline in the ability to discriminate between emotional states in MS (Cotter et al., 2016).

Moreover, little is still known regarding as to whether ToM impairment is related to disease duration and level of disability. Most works included subjects with a low level of disability and at an early stage of the disease, revealing the presence of a deficit already since the early phases of the disease (Kraemer et al., 2013). Furthermore, studies also reported greater impairment of ToM abilities in progressive (Pr; such as Secondary Progressive MS and Primary Progressive MS) compared to relapsing-remitting (RR) MS (Berneiser et al., 2014; Radlak, 2014; Dulau et al., 2017). However, recently, one work reported no difference in ToM performance between MS phenotypes (Dulau, 2014). Hence, the relation between ToM impairment and clinical course is not yet well understood. Generally, there are few studies that have characterized ToM impairment in MS populations and results are conflicting. Literature meta-analysis and reviews agree that this topic needs more investigation (Bora et al., 2016; Cotter et al., 2016; Chalah and Ayache, 2017).

The role played by deficits in cognitive functions on ToM impairment is another point of debate (Cabinio et al., 2015; Chalah et al., 2017; Ciampi et al., 2018; Neuhaus et al., 2018; Wade et al., 2018). This aspect is particularly interesting considering that social cognition involves specific networks that overlap and are connected with areas recruited for other cognitive functions (Henry et al., 2016). Some studies highlighted that ToM impairment is independent from cognitive domains mainly affected in MS, e.g., executive functions (Neuhaus et al., 2018). On the other hand, Dulau et al. (2017) have reported a link between impairment in cognitive functions and ToM abilities. Moreover, differences recently highlighted in cognitive impairment profile between RR and progressive MS (Johnen et al., 2017) have also to be considered whether it is supported that ToM is related to cognitive functions.

Finally, although extensive data exist for cognitive functions in MS, the link between ToM and QoL has received relatively little attention. It is widely acknowledged that a deficit in ToM has a crucial influence on QoL, especially in chronic conditions. In fact, ToM, such as a crucial component of social cognition, promotes the continuity of social activities during the entire life span of patients, preventing also depressive symptomatology (Chiaravalloti and DeLuca, 2008). Accordingly, literature reports several works investigating the role of ToM on QoL in different chronic pathologies (Bodden et al., 2010b; Urbach et al., 2013). For what concerns the impact of ToM on QoL specifically in MS condition, only few studies on this topic reported QoL measures and even less reporting meaningful results. This is unexpected, since good interpersonal relationships and social support have been highlighted as important factors increasing QoL in MS (Shwartz and Frohner, 2005). In addition, as concluded by Raimo et al. (2017), ToM ability is extremely useful in everyday life and social environment. Moreover, works on the topic reported conflicting results: Phillips et al. (2011) identified social cognition deficit as a main predictor of QoL in MS patients (Cotter et al., 2016). Instead, Ciampi et al. (2018) reported no correlation between ToM impairment and QoL. Given that QoL of persons who manage chronic pathologies is an urgent focus of intervention, its relationship with SC is non-negligible and a better understanding of such deficits is crucial to improving patients’ QoL.

The main aim of this study was to assess the relation between ToM, clinical variables (disease duration and disability level) and neuropsychological profile in a cohort of adult patients with different clinical phenotypes (Relapsing Remitting versus Progressive). Hence, the specific aims consisted of determining: [1] How (affective and cognitive) ToM impairment occurs in relation with clinical phenotypes: Relapsing Remitting MS versus Progressive MS versus healthy controls; [2] if ToM impairment in MS is secondary to the cognitive deficits; [3] if deficit in ToM competence impacts QoL.

On the light of the recent literature on the topic, we hypothesized that Progressive MS phenotype shows a major impairment in ToM abilities due to its neurodegenerative pathogenic component, both in cognitive and affective parts of ToM; in particular, according to the more severe cognitive impairment found in Progressive than Relapsing Remitting MS, we expected to find a relation between cognitive functions level and ToM performance. Also, given the strong contribution of mentalizing abilities in social relationships in daily life, we hypothesized to observe a relation between QoL and ToM.

## Materials and Methods

### Participants

A total of 68 subjects were enrolled in the study: 42 individuals with MS and 26 healthy controls. Patients with MS were consecutively recruited and screened at Don Carlo Gnocchi Foundation, IRCCS, Milan (Italy). Inclusion criteria were: (1) diagnosis of MS following McDonald revised diagnosis criteria (Polman et al., 2011), (2) age < 75, (3) years of education ≥ 5, (4) no change of pharmacological treatment in the 3 months before the enrollment (5) no clinical relapses or use of steroid treatment during 4 weeks before the enrollment (6) provided informed consent for study participation. Exclusion criteria were: (1) history of nervous system disorders different from MS (2) unstable psychiatric illness, such as psychosis or major depression (3) severe disability with Expanded Disability Status Scale (EDSS, Kurtzke, 1983) score > 8 (4) moderate to severe cognitive impairment (Mini Mental State Examination < 24) (5) medication or comorbidities that may affect cognition (6) severe visual or/and hearing impairment that did not allow to perform evaluation battery.

A neurologist collected medical history and performed physical examination of all MS patients. Based on Polman’s Criteria (Polman et al., 2011) MS were defined Relapsing Remitting MS (*N* = 26) or Progressive MS (*N* = 16). The group of 16 Progressive MS comprised subjects with Secondary Progressive MS (*N* = 8) and Primary Progressive MS (*N* = 8). For all MS subjects EDSS and disease duration were collected.

26 healthy subjects from Don Carlo Gnocchi Foundation of Milan were also recruited. They were personnel of the clinic or healthy adults accessing to the clinic such as volunteers and caregivers. The healthy participants were group-matched for age, sex, and years of education with the MS group. Inclusion criteria included not having any known neurological diseases or psychiatric condition, a global cognitive function in normal value for age and education, and do not taking any drugs that affect cognitive functions.

The study was approved by Universitá Cattolica del Sacro Cuore Ethical Committee. All participants received the information sheet and signed the written informed consent.

### ToM Instruments

The following ToM tasks evaluating both cognitive and affective components of mentalization were administered to all participants of the study:

[1] Reading the Mind from the Eyes (RMET, Baron-Cohen et al., 2001): an affective ToM test (Duval et al., 2011; Laisney et al., 2013). The task presents to the subject 36 visual stimuli, such as photographs of actors’ eyes expressing a certain basic or complex emotion and requires him/her to choose the right mental state among four alternatives. Total score ranges from 0 to 36. We adopted Gender test as the control task: we considered RMET data only whether subject correctly performed the Gender test;
[2] Strange Stories (SS; Happé, 1994; Mazzola and Camaioni, 2002; Liverta Sempio et al., 2005): a selection of the Italian version of Strange Stories, 4 mental and 4 physical (control stories) content stories, were administered as an advanced ToM task.
Subjects were presented orally stories in which a character says something not literally true and they were asked to explain reasons of the specific behavior of the agent. Each story was presented once. Mentalistic stories chosen in the current study require an understanding of mental attribution in different social situations: conflicting emotions, misunderstanding, white lie and double bluff. Each story score ranges from 0 to 2: a score of 0 is attributed when subject answers something incoherent with the story, a score of 1 corresponds to a factual response, while 2 points score are given to a correct mentalistic answer. The highest total score is 8 both for ToM and physical content stories. Although this task is mainly presented in literature as a cognitive ToM tool (Poletti et al., 2012), a consensus on the cognitive or affective ToM nature of this test has not yet reached; in fact, while some stories lead to reason on characters’ thoughts, others are focuses on characters’ emotions. For the purpose of this study, we chose to consider not only Strange Story total score (0–8 range score), but also single-story scores (0–2 range score) in order to compare thoughts understanding (cognitive ToM), such as double bluff, misunderstanding and white lie story, with emotion reasoning (affective ToM), in particular conflicting emotions story.
[3] Faux pas (Baron-Cohen et al., 1999): a selection of the Italian version of the test, specifically 4 faux pas and 4 matched control stories, were included in the evaluation battery as an advanced ToM test. Subjects were asked to listen to stories, each story were presented orally and once by the researcher, in which a faux pas happened and to answer six questions. Questions require the faux pas detection, the identification of the person who does something awkward, the description and comprehension of the content of the faux pas, the detection of false belief and the answer to an empathy question. In case the subject incorrectly answers the first question, the other questions are not administered. The total score for each story ranges from 0 to 6, with a maximum total score of 24. In addition to the total score of the test, we focused on cognitive and affective items in order to compare cognitive and affective ToM performance: in particular, we reported score of the intentionality question (cognitive ToM; score range 0–4) and score of the emotional attribution question (affective ToM; score range 0–4).
[4] Movie for the Assessment of Social Cognition (MASC; Dziobek et al., 2006; Fossati et al., 2018): the Italian version of the test were administered as a naturalistic evaluation of ToM abilities. The test consists in watching a 15-min video clip representing an ecologic situation in which four friends meet to have a dinner together. Video is interrupted several times by a multiple-choice question on emotions (17 item), thoughts (7 item) and intentions (18 item) of the different characters. More specifically, each question reports 4 alternative responses including one correct mentalistic answer and three incorrect ones: a non-mentalistic (missing), a over-mentalizing and a under-mentalizing option. The task total score ranges from 0 to 42 and in addition yields a score corresponding to the total number of mistakes due to over-mentalizing (0–42 range), under-mentalizing (0–42 range) and no mental state attribution (0–42 range). Following the purpose of the present study, we calculated scores separately for emotions (affective ToM; 0–17 range), thoughts and intentions items (cognitive ToM; respectively, 0–7 and 0–18 range): in particular, we reported the sum of total right responses, sum of mistakes due to over-mentalizing, sum of mistakes due under-mentalizing, and sum of mistakes due no mental state attribution for each of them.

### Neuropsychological Assessment

All participants underwent Montreal Cognitive Assessment (MoCA) for global cognitive function evaluation (Santangelo et al., 2015). The MS group was also assessed with the Brief Repeatable Battery of Neuropsychological Test (BRB-NT; Rao, 1990) for an in-depth assessment of memory and executive functions including the following neuropsychological tests: [1] Selecting Reminding Test – Long Term Storage (SRT-LTS), evaluating verbal immediate recall (cut-off :23.3); [2] Selective Reminding Test – Consistent Long Term Retrieval (SRT-CLTR), evaluating verbal memory storage (cut-off: 15.5); [3] 10/36 Spatial Recall Test (SPART), a measure of visuo-spatial memory (cut-off: 12.7); [4] Symbol Digit Modalities Test (SDMT), assessing attention and speed of processing (cut-off: 37.9); [5] Paced Auditory Serial Addition Test (PASAT 2/ PASAT 3), evaluating divided attention (cut-off: 17.1/28.4); [6] Delayed Recall of the Selective Reminding Test (SRT-D), assessing verbal delayed recall (cut-off: 4.9); [7] Delayed Recall of the 10/36 Spatial Recall Test (SPART-D), measuring visuo-spatial delayed recall (cut-off: 3.6); [8] Word List Generation (WLG), a measure of the access to lexicon through semantic category (cut-off: 17.0).

### Psycho-Behavioral and QoL Assessment

The entire sample filled in the following behavioral questionnaires administered in computerized form:

[1] Beck Depression Inventory-II (BDI-II; Beck et al., 1996; Ghisi et al., 2006): a measure of clinical depression (cut-off < 16). The scale allows to extract two subscales: cognitive symptoms and non-cognitive symptoms.
[2] State-Trait Anxiety Inventory – Y1 (STAI-Y1; Santangelo et al., 2016): a test evaluating the presence of clinical level of anxiety state (cut off 39–40). Total score ranges from 20 to 80.
[3] PARADISE 24 (Cieza et al., 2015): a questionnaire measuring the impact of brain disorders on people’s lives. Total score ranges from 0 (no impact) to 100 (extreme impact) (Giovannetti et al., 2016). The questionnaire was filled in only by participants with MS.
[4] Multiple Sclerosis Quality of Life 54 questionnaire (MSQOL54; Solari et al., 1999): a measure of QoL in MS sample including all items of the Short form 36 health survey questionnaire (SF36; Jenkinson et al., 1993) and of the MS18 module. Only items of SF12 were administered to healthy control subjects. SF36 allows to extract two Health total scores: Physical and Mental Health. Specifically, following SF36 model, Physical Health scale was calculated on Physical Functioning, Role-Physical, Bodily Pain and General Health sub-scales, while Mental Health was calculated on Vitality, Social Functioning, Role-Emotional and Mental Health sub-scales.

### Procedure

Neuropsychological functioning, behavioral profile and ToM ability were evaluated in a dedicated session by a psychologist.

The evaluation session was individual and took place in a quiet room. It was divided in two parts, lasting about 1 h each. The order of the two evaluation sections was balanced.

The session included a classic paper and pencil neuropsychological evaluation, the administration of ToM tests and behavioral questionnaires. Part of the assessment was carried out via computer.

Healthy participants took part in the same session with a reduced neuropsychological evaluation.

### Statistical Analysis

Data was analyzed with Statistical Package for Social Sciences (SPSS, IBM Corporation), version 24.

To test how (affective and cognitive) ToM impairment occurs in relation to clinical phenotypes, a statistical comparison between MS and healthy controls was performed. Normal distribution of variables was checked through normality test on variables residuals. According to this test, parametric (independent *t*-test) or non-parametric analysis (Mann–Whitney U) was performed for the comparison between the two groups (MS and healthy controls). Also, Multiple General Linear Model was run to compare cognitive and affective ToM among the three groups. SS Physical stories, Faux Pas control stories and BDI-II were included in the Model as covariates.

To test whether ToM impairment in MS comes from the cognitive deficit we firstly run correlation analysis including all MS sample between ToM performance and neuropsychological scores. Then, Linear Regression was calculated to investigate predictive relations between ToM performance and neuropsychological measures in all MS group.

To test whether a low performance of ToM is related to a lower QoL, General Linear Model was performed with QoL scores as within-subject factor and ToM scores as between-subject factor. Results were considered statistically significant at *p* < 0.05.

## Results

### Participants

Table 1 presents demographic characteristics of MS and group comparison results. Control group matched MS groups for age, gender and education. In line with literature, MS group showed to be more depressed than healthy controls, specifically due to no cognitive symptoms. Also, we found a statistically significant difference between healthy controls and MS groups in Physical Health Composite Score (PHCS). This result was significant both observing the whole MS group and focusing on different MS phenotypes. In particular, comparing the three groups, Kruskal–Wallis test reported that both RR and Pr MS groups had a lower PHCS than healthy controls. Table 2 reports neuropsychological test results. In particular, a statistically significant difference between MS and healthy control groups in MoCA total score was also observed, with a worse performance in MS subjects than healthy control group, albeit it remained over Santangelo’s correction cut-off (< 15.05). Specifically, One-way ANOVA with MoCA score as dependent variable and phenotype as independent variable showed a main effect of phenotype and Bonferroni *post hoc* test reported a statistically significant difference only between PrMS and healthy control group. BRB-NT assessment reported a mean total score over cut-off for each sub-scale in MS sample.

**Table 1:** Demographic characteristics and group comparison results.

	HCD *N* = 26	all MS *N* = 42	MS/HC comparison	*p*	RRMS *N* = 26	PrMS *N* = 16	RRMS/PrMS/HC comparison	*p*
Gender [M:F]	7:19	18:24	3.76^&^	-	10:16	8:8	2.94^&^	-
Age [M, sd]	51.35,12.42	52.38,10.31	-0.37^§^	-	50.42,10.09	55.56,10.18	1.96^£^	-
Years of education [M, sd]	14.88,4.93	12.76,4.21	1.89^§^	-	12.42,2.86	13.31,5.86	3.42^£^	-
EDSS[Median, range]	-	6,1–8	-	-	6, 1–7	7, 6–8	-	-
Disease duration [M, sd]	-	21.24,10.94	-	-	21.08,11.86	21.50,9.60	-	-
**BDI-II** [M, sd]	5.00, 4.89	8.86,8.69	-**2.13^∧^**	**0.04**	9.38, 8.98	8.00, 7.44	4.06^£^	-
Cognitive symptoms	1,42, 2,32	2,55,3,42	-1.48^∧^	-	2.85, 3.73	2.06, 2.91	2.85^£^	-
**No cognitive symptoms**	3.58, 3.61	6.31,5.30	-**2.31^∧^**	**0.03**	6.54, 5.59	5.94, 4.93	4.98^£^	-
STAI-Y1[M, sd]	13.19, 3.20	15.67,6.03	-1.93^∧^	-	15.77,5.69	15.50,6.74	3.14^£^	-
PARADISE [M, sd]	-	50.03,16.08	-	-	50.66,16.15	49.00,16.44	-	-
MSQOL54 [M, ds]								
Physical health		55.95, 17.32	-	-	58.70, 18.54	51.47, 14.58	-	-
Mental health		64.20, 20.76	-	-	65.06, 21.94	62.81, 19.28	-	-
SF12 [M, sd]								
**Physical health**	51.41, 6,53	34.87, 9.61	**103.00^∧^**	**0.000**	36.34, 10.58	32.47, 7.47	**32.39**°	**0.000**
Mental health	49.46, 10.04	51.10, 10.71	482.50^∧^	-	51.10, 10.70	51.11, 11.05	0.64°	-

*BDI-II, beck depression inventory, HC, healthy controls; M, mean; MS, multiple sclerosis; MSQOL54, multiple sclerosis quality of life 54; RRMS, relapsing remitting multiple sclerosis; PrMS, progressive multiple sclerosis; sd, standard deviation; STAI -Y1, State-Trait Anxiety Inventory – Y1. ^§^*T*-test for independent samples was performed; ^&^Chi-squared test was performed; °Kruskal–Wallis test was performed;^∧^Mann–Whitney test was performed; ^£^One-way ANOVA was performed. Statistically significant results are reported in bold*.

**Table 2:** Neuropsychological test and group comparison results.

	Score Range	HC *N* = 26	all MS *N* = 42	MS/HC comparison	*p*	RRMS *N* = 26	PrMS *N* = 16	RRMS/PrMS/HC comparison	*p*
**MoCA** [M, sd]	0–30	26.61, 2.10	22.92, 3.30	**2.40^§^**	**0.007**	24.96, 3.56	22.87, 3.28	**5.32^£^**	**0.008**
BRB-NT [M, sd]									
SRT-LTS [M, sd]	0–72	-	35.85, 14.80	**-**	-	37.31, 14.02	33.47, 16.16	**-**	
SRT-CLTR [M, ds]	0–72	-	29.37, 15.56	**-**	-	30.56, 16.52	27.44, 14.16	**-**	
SPART [M, sd]	0–30	-	19.47, 5.74	**-**	-	19.42, 5.35	19.56, 6.51	**-**	
SDMT [M, sd]	0–110	-	39.72, 16.05	**-**	-	41.55, 17.63	36.75, 13.08	**-**	
PASAT2 [M, sd]	0–60	-	25.76, 11.75	**-**	-	35.11, 15.22	31.18, 11.45	**-**	
PASAT3 [M, sd]	0–60	-	33.62, 13.89	**-**	-	28.13, 13.43	21.93, 7.16	**-**	
SRT-D [M, sd]	0–12	-	6.84, 2.48	**-**	-	7.08, 2.56	6.46, 2.37	**-**	
SPART-D [M, sd]	0–10	-	6.88, 2.12	**-**	-	7.11, 2.54	6.52, 1.55	**-**	
WLG [M, sd]	0-∞	-	21.29, 5.97	**-**	-	21.09, 7.08	21.63, 3.72		

*BRB-NT, brief repeatable battery of neuropsychological test; MoCA, montreal cognitive assessment; PASAT 2, 2 s paced auditory serial addition test; PASAT 3, 3 s paced auditory serial addition test; SDMT, symbol digit modalities test; SPART, 10/36 spatial recall test; SPART-D, delayed recall of the 10/36 spatial recall test; SRT-D, delayed recall of the selective reminding test; SRT-CLTR, selective reminding test – consistent long term retrieval; SRT-LTS, selecting reminding test – long term storage; WLG, word list generation. ^§^T-test for independent samples was performed; ^£^One-way ANOVA was performed. Statistically significant results are reported in bold*.

### AIM 1: How Does Affective and Cognitive ToM Impairment Occur in Relation With Clinical Phenotype?

Table 3 reports ToM tasks groups performance and comparison results. In general, we found statistically significant groups differences in SS and Faux pas total score. Specifically, we observed that both MS groups showed a significant lower SS global score than healthy controls; while only Pr MS group differed significantly from healthy controls in Faux-pas total score. Considering cognitive and affective items of SS and Faux-pas, results highlighted a significant groups differences in cognitive items only: MS groups showed lower scores than healthy controls in double bluff, white lie and misunderstanding, but not contrasting emotions stories, as well as they reported lower score in intentionality item, but not in emotion item of Faux pas. In line with total score results, both RR and Pr MS groups differed from healthy controls in SS cognitive stories, while only Pr MS group had a lower score than healthy controls in intentionality question of Faux pas. Moreover, we found a statistically significant difference between the three groups in over-mentalizing errors for affective items of MASC. Mann–Whitney and Bonferroni *post hoc* test showed that the difference was explained only by RR and Pr MS groups, with a higher score of Pr MS group.

**Table 3:** Theory of mind assessment and group comparison results.

	HC *N* = 26	MS *N* = 42	MS/HC comparison	*p*	Partial η^2^, observed power	RR *N* = 26	Pr *N* = 16	RR /Pr/HC comparison	*p*	Partial η^2^, observed power	Bonferroni’s *post hoc*
**ToM score [M, sd]**											
**SS**	6.00, 1.2	4.38, 1.38	**200.50^∧^**	**0.000**	**0.27, 1.00**	4.42, 1.42	4.31, 1.35	**19.77°**	**0.000**	**0.27, 0.99**	MS < HC
**SS**_physicalstories_	6.12, 1.03	5.31, 1.66	398.00^∧^	-	-	5.81, 1.47	4.50, 1.67	**10.48°**	**0.005**	**0.18, 0.92**	Pr < HC, RR
**FP**	17.23, 4.36	14.19, 6.00	**2.24^§^**	**0.028**	**0.07, 0,60**	15.42, 4.43	12.19, 7.68	**4.43^£^**	**0.016**	**0.12, 0.74**	Pr < HC
**FP**_controlstories_	3.58, 0.50	3.64, 0.73	-0.41^§^	-	-	3.65, 0.48	3.63, 1.02	0.09^§^	-	-	-
MASC _totalscore_	29.85, 4.58	29.93, 6.01	-0.06^§^	-	-	30.27, 2.97	29.38, 6.18	0.13^£^	-	-	-
_Missingmentalizing_	3.73, 2.34	4.05, 3.27	-0.43^§^	-	-	3.92, 2.81	4.25, 3.99	0.15^£^	-	-	-
_Under-mentalizing_	5.31, 2.29	5.64, 2.94	-0.49^§^	-	-	5.58, 2.98	5.75, 2.95	0.14^£^	-	-	-
_Over-mentalizing_	4.96, 2.64	4.48, 2.33	0.79^§^	-	-	3.88, 2.20	5.44, 2.28	2.41^£^	-	-	-
**Cognitive ToM [M, sd]**											
**SS**_Doublebluff_	0.66, 0.43	29.80	**348.50^∧^**	**0.010**	**0.09, 0.72**	0.37, 0.45	0.40,0.45	**6.60°**	**0.037**	**0.09, 0.61**	RR < HC
**SS**_Whitelie_	1.03, 0.15	29.37	**330.50^∧^**	**0.002**	**0.12, 0.84**	0.84, 0.34	0.76,0.35	**10.97°**	**0.004**	**0.13, 0.78**	MS < HC
**SS**_Misunderstanding_	0.81, 0.23	30.80	**390.50^∧^**	**0.026**	**0.08, 0.68**	0.65, 0.27	0.69,0.19	4.99°	-	-	-
**FP**_Intention_	2.31, 0.74	1.67, 1.12	**2.85**^§^	**0.006**	**0.09, 0.72**	1.73, 0.92	1.56, 1.41	**3.46**^£^	**0.037**	**0.10, 0.63**	Pr < HC
MASC _Thoughts_	5.46, 1.27	5.26, 1.41	0.59^§^	-	-	5.12, 1.34	5.50, 1.46	0.56^£^	-	-	-
_Missingmentalizing_	0.61, 0.64	34.48	545.00^∧^	-	-	0.65, 0.69	0.69,1.01	0.12°	-	-	-
_Under-mentalizing_	0.85, 0.73	33.55	506.00^∧^	-	-	0.96, 1.22	0.75,1.12	0.81°	-	-	-
_Over-mentalizing_	1.04, 0.92	36.36	468.00^∧^	-	-	1.27,0.72	1.13,0.62	1.75°	-	-	-
MASC _Intention_	13.08, 2.06	13.31, 3.29	-0.36^§^	-	-	13.73, 2.97	12.63, 3.76	0.78^£^	-	-	-
_Missingmentalizing_	1.58, 1.27	33.46	502.50^∧^	-	-	1.50, 1.36	1.62, 2.09	0.51°	-	-	-
_Under-mentalizing_	1.62, 1.10	34.75	535.50^∧^	-	-	1.50, 1.33	2.06, 1.69	1.12°	-	-	-
_Over-mentalizing_	1.77, 1.42	32.61	466.50^∧^	-	-	1.23, 1.39	1.69, 1.25	2.51°	-	-	-
**Affective ToM [M, sd]**											
SS_Emotions_	0.76, 0.27	34.10	529.00^∧^	-	-	0.79, 0.26	0.66, 0.25	2.51°	-	-	-
FP_Emotion_	2.85, 0.97	2.40, 1.17	1.61^§^	-	-	2.62, 98	2.06, 1.39	2.62^£^	-	-	-
RMET	25.04, 3.30	23.83, 3.41	1.43^§^	-	-	24.12, 2.75	23.38, 4.35	1.25^£^	-	-	-
MASC_Affective_	10.50,1.90	10.40, 2.14	0.19^§^	-	-	10.77, 2.23	9.81, 1.90	1.11^£^	-	-	-
_Missingmentalizing_	1.54, 1.36	35.68	496.50^∧^	-	-	1.77, 1.48	1.94, 1.77	0.44°	-	-	-
_Under-mentalizing_	2.85, 1.46	35.92	486.50^∧^	-	-	3.08, 1.38	2.88, 1.26	0.90°	-	-	-
_Over-mentalizing_	2.12, 1.42	32.70	470.50^∧^	-	-	1.31, 1.01	2.50, 1.32	**9.14°**	**0.010**	**0.14, 0.83**	RR < Pr

*HC, healthy controls; FP, faux pas, M, mean; MASC, movie for the assessment of social cognition; MS, multiple sclerosis; RRMS, relapsing remitting multiple sclerosis; PrMS, progressive multiple sclerosis; RMET, reading the mind from the eyes test; sd, standard deviation. ^§^*T*-test for independent samples was performed; ^∧^Mann–Whitney test was performed; ^£^One-way ANOVA was performed; °Kruskal–Wallis test was performed. Statistically significant results are reported in bold*.

A multivariate General Linear Model (GLM) was performed with 4 levels of SS (contrasting emotions, double bluff, misunderstanding and white lie) as *within-subject* factor and 3 levels of phenotype (healthy controls, RR MS and Pr MS) as *between-subject* factor. Results showed a main effect of phenotype for double bluff (*F*_2,65_ = 3.32, *p* = 0.042, partial-η^2^ = 0.09, observed power = 0.61) and white lie (*F*_2,65_ = 4.84, *p* = 0.011, partial-η^2^ = 0.13, observed power = 0.78), and a trend of phenotype for misunderstanding (*F*_2,65_ = 3.09, *p* = 0.05, partial-η^2^ = 0.09, observed power = 0.58). The analysis was run also with SS physical stories and BDI-II as covariates: results continued to show a main effect of phenotype for white lie (*F*_2,65_ = 5.58, *p* = 0.006, partial-η^2^ = 0.15, observed power = 0.84), a trend for double bluff (*F*_2,65_ = 3.07, *p* = 0.053, partial-η^2^ = 0.09, observed power = 0.57), but not anymore for misunderstanding (*F*_2,65_ = 2.18, *p* = 0.122, partial-η^2^ = 0.06, observed power = 0.43).

Also, a multivariate GLM was performed with 2 level of Faux pas (intentionality and emotion) as *within-subject* factor and 3 level of phenotype (healthy controls, RR MS and Pr MS) as *between-subject* factor. We observed a main effect of phenotype only for intentionality (*F*_2,65_ = 3.46, *p* = 0.037, partial-η^2^ = 0.10, observed power = 0.63). We repeated analysis with BDI-II as covariates and we didn’t find any statistically significant effect of this variable.

### AIM 2: Is ToM Impairment Secondary to Cognitive Deficit in MS?

We found a statistically significant correlation between SS and MoCA (*r* = 0.394, *p* = 0.010), SPART (*r* = 0.496, *p* = 0.001), SDMT (*r* = 0.310, *p* = 0.046), PASAT3 (*r* = 0.355, *p* = 0.021), PASAT2 (*r* = 0.306, *p* = 0.049), SPART-D (*r* = 0.384, *p* = 0.012) and WLG (*r* = 0.343, *p* = 0.026). Results also showed that Faux pas performance statistically significant correlated with SPART (*r* = 0.397, *p* = 0.009), SDMT (*r* = 0.312, *p* = 0.044), PASAT3 (*r* = 0.437, *p* = 0.004), SRT (*r* = 0.438, *p* = 0.004) and SPART-D (*r* = 0.336, *p* = 0.030). Finally, we found a statistically significant correlation between RMET and SPART (*r* = 0.394, *p* = 0.010).

To verify whether executive functions have a predictive effect on ToM performance in all MS sample, regression analyses were performed introducing SDMT and PASAT3 on (1) SS performance and (2) Faux pas (dependent variable), alongside phenotype (RR, Pr). These predictors were entered, hierarchically, in two steps: model 1: SDMT, PASAT3; model 2: SDMT, PASAT3, phenotype.

Results showed that model 1 tended to be statistically significant in predicting SS (Model 1: *F*_2,41_ = 3.24, *p* = 0.05, *R*^2^ = 0.14, *R*^2^_adjusted_ = 0.10), while both models were statistically significant in predicting Faux pas (Model 1: *F*_2,41_ = 4.61, *p* = 0.016, *R*^2^ = 0.19, *R*^2^_adjusted_ = 0.15; Model 2 = *F*_2,41_ = 3.87, *p* = 0.017, *R*^2^ = 0.23, *R*^2^_adjusted_ = 0.17). Statistical details are listed in Table 4.

**Table 4:** Regression analysis models of executive functions on ToM performance in all MS sample.

		Model 1			Model 2		
	Predictors	B	SE(B)	β	B	SE(B)	β
(1) SS	SDMT	0.02	0.02	0.20	0.02	0.02	0.20
	PASAT3	0.02	0.02	0.21	0.02	0.02	0.21
	Phenotype_ dummy_				0.05	0.43	0.02
	R2		0.14			0.14	
	F for change in R2		**3.24^∧^**			2.11	
	*P*		**0.05**			-	
	N	42	42	42	42	42	42
(2) FP	SDMT	-0.00	0.08	-0.00	-0.01	0.08	-0.02
	PASAT3	0.19	0.09	0.44	0.19	0.09	0.42
	Phenotype_ dummy_				-2.55	1.76	-0.21
	R2		0.19			0.23	
	F for change in R2		**4.61**			**3.87**	
	*P*		**0.016**			**0.017**	
	N	42	42	42	42	42	42

*FP, faux pas; SS, strange stories; ^∧^tendency to significance. Statistically significant results are reported in bold*.

### Does Deficit in ToM Competence Impact QoL in MS?

To test whether different level of ToM differed significantly in level of QoL, we performed GLM models with MSQOL54 scores as *within-subject* factor and ToM performance as *between-subject* factor.

We observed a main effect of SS contrasting emotion story on both Physical Health (*F*_2,42_ = 3.65, *p* = 0.019, partial-η^2^ = 0.16, observed power = 0.64) and Mental Health (*F*_2,42_ = 4.25, *p* = 0.007, partial-η^2^ = 0.18, observed power = 0.71).

## Discussion

Social cognition deficit in neurodegenerative chronic conditions is increasingly being investigated with respect to its impact on daily life (An and Jang, 2018). Recently, research highlighted the presence of ToM impairment in MS population (Henry et al., 2011). However, it is still not clear which components of ToM are damaged as well as the role of clinical profile and cognitive functions. The present study aimed to evaluate ToM in MS, considering both affective and cognitive components, and its relationship with clinical and neurocognitive profile. Also, the impact of ToM deficit on QoL was investigated.

In our study MS subjects failed in ToM tasks described in literature as advanced tests with a considerable level of complexity (Banerjee, 2005; Devine and Hughes, 2016). In particular, we found a stronger impairment in cognitive mentalizing ability of Pr MS, who fell both in SS and Faux-pas cognitive items, than RR MS, who reported only a minor performance in SS cognitive items. The only exception interested MASC cognitive items, in which our MS patients did not show a damage. With respect of this latter result, the dynamic feature of the stimuli distinguishes MASC from other ToM tasks, as it allows patients to experience stimuli showing all body agent’s figure in movement. This issue, combined with the presence of the plot underlining characters’ exchanges, could have acted as a scaffolding element, favoring compensatory arrangements. In fact, some studies have shown that reading fiction supports ToM development (Kidd and Castano, 2013; Pino and Mazza, 2016); it is therefore plausible that even a cinematographic plot may have some positive influence on the understanding of mental states. With regards to dynamism, some studies investigated the activation of different neural pathways depending on static, generally less ecologic, or dynamic features of emotional stimuli (Di Dio et al., 2016). In particular, the distribution of neural activations in response to biological motion of facial expression differs from static representation of emotional expressions (Kilts et al., 2003; Trautmann et al., 2009). The fact that our sample showed a cognitive ToM impairment in all tasks except for the one with stimuli resembling to real life situation, considered indeed more ecological, lead us to hypothesize that mentalizing damage in MS could not have a strong functional real-life implication and reveals itself only in situations which individual is not familiar with.

Focusing on affective ToM, the fact that affective items were not impaired leads one to assume that the difficulty in MS is limited to the comprehension of thoughts and intentions, but not emotions. Taken together, these results suggest dissociative damage of cognitive and affective ToM in this population. Indeed, in both SS and Faux pas task we found that affective comprehension was maintained. Moreover, RMET performance was comparable to the healthy population. This latter result was unexpected and counter-trend, since facial emotions identification has consistently been shown impaired in MS patients (Henry et al., 2009; Banati et al., 2010). However, to our knowledge, this is the first study investigating ToM in individuals with a long story of MS, such as 20 years of disease history, with no cognitive impairment. Moreover, also the mean age of our groups was major than previous works and, given that it is demonstrated in literature that age is negatively correlated with RMET performance (Fischer et al., 2017), it is plausible to assume that MS performance to affective ToM did not differ from a group of age-matched healthy subjects. In general, the dissociation of ToM components (cognitive versus affective) in clinical conditions has been documented in the literature (Shamay-Tsoory et al., 2007; Poletti et al., 2012). However, to our knowledge, only two studies addressed this issue in MS (Raimo et al., 2017; Roca et al., 2014) with contrasting results. The work of Raimo et al. (2017) reported no dissociation in ToM in early stages of the disease, while, on the contrary, Roca et al. (2014) demonstrated the presence of only cognitive ToM deficit in early MS. Our results, including a MS population with a moderate disability (EDSS, median = 6, range = 1–8) and a quite long disease story (disease duration, mean 21.24), confirmed Roca et al. (2014) evidence: cognitive and not affective ToM is impaired in MS. Our findings, in accordance with the results of Roca et al. (2014), lead to the assumption that this dissociation lasts from early until moderate stages of the disease. Also, we can hypothesize that the cognitive component of ToM is impaired from the beginning of the disease, as Roca et al. (2014) reported, while affective ToM impairment occurs in a second phase. Only a longitudinal study can disentangle this question.

Analyzing the relation between cognitive performance and mentalizing, data showed significant predictive effects of neuropsychological measures on ToM, especially on advanced ToM tasks, requiring a higher cognitive load, in which MS sample encountered more difficulties. Interestingly, we found that executive function and phenotype only predicted faux pas performance, while only executive function and not phenotype predicted SS. This data, in accordance with some previous studies (Chalah et al., 2017; Dulau et al., 2017) suggests that cognitive level plays a role, a predictive effect, on ToM impairment in patients with MS. The fact that specific brain regions underlying social cognition are also involved or overlap with areas serving for other non-social cognitive functions (Henry et al., 2016; Wade et al., 2018) could partly explain the strong relation between cognitive functions and ToM found in the present study. Also, this result is in line with studies attesting diverse cognitive factors associated with disease progression (Chiaravalloti and DeLuca, 2008) and not only to the cerebral dissociations of cognitive and affective routes of ToM networks. In line with this evidence, we reported a predictive role specific for executive functions only on ToM task in which MS remitting and progressive phenotype differed. Findings of Ciampi et al. (2018) support the direct correlation of social cognition performance with traditional cognitive domains in progressive MS. Accordingly, we reported a major damage of ToM in progressive typologies of MS, in line with evidence of works of Berneiser et al. (2014), Dulau et al. (2017), and Radlak (2014).

In addition, the present study investigated not only the relationship between cognitive functions and ToM, but also between QoL and ToM. Results suggested a significant implication of affective ToM on different domains of QoL, which is in line with evidence of works demonstrating the crucial role of social cognition in supporting QoL (Maat et al., 2012). Specifically, we found that subjects with a lower score of strange stories on emotions differed in level of physical and mental QoL. This finding supports the central role of social mind abilities in impacting QoL of people with MS. On the other hand, this result revealed us that only affective and not cognitive ToM is related with QoL. As in our sample affective ToM was preserved, we can assume that the implications of mentalizing impairment in everyday life do not consistently influence MS QoL.

Our study is not without limitations. First of all, due to the limited number of cases of secondary and primary progressive MS patients enrolled in the study, we were only able to investigate RRMS and Progressive MS groups. In addition, the fact that healthy subjects were not administrated all selected neuropsychological battery, but only MoCA, could have moved the attention from possible informative data for the discussion of results. Another limitation of the present work consists in the lack of an IQ test, not allowing to investigate relations between mentalizing and intelligence in MS patients. Also, due to the cross-sectional nature of our study, we are limited in our ability to investigate causal effects between the investigated domains. Future longitudinal studies and cross-sectional studies with wider samples are needed.

In general, the present study confirmed our first hypothesis reporting a major ToM impairment in progressive phenotype than remitting MS. Unexpectedly, we found a dissociation between cognitive and affective ToM, with a specific deficit of cognitive ToM that still not affect more real-life resembling stimuli. Results were also aligned with our second hypothesis: cognitive functions predict advanced mentalizing abilities in MS. Finally, a relation between QoL and ToM, specifically affective component of ToM, was demonstrated.

## Conclusion

Cognitive but not affective components of ToM are impaired in MS with a long disease duration. Both clinical and cognitive variables have a substantial role: PrMS appears more damaged in cognitive ToM and cognitive functions predict ToM performance. In this work we focused on the evaluation of a specific domain of social cognition in MS, such as ToM. However, future contributions may highlight on the whole social cognition assessment (Labbé et al., 2018), including also affective empathy, social behavior and social perception. Given the impact of ToM on QoL in MS, clinical practice should include psychosocial evaluation in MS screening with the future purpose to offer patients multidimensional interventions including social cognition rehabilitation activities for this population.

## Author Contributions

DM, AM, and FB contributed to the conception of the work. DM, FB, AM, and SI contributed to the analysis and interpretation of the data. SI drafted the manuscript. All authors revised and approved the final manuscript.

## Conflict of Interest Statement

The authors declare that the research was conducted in the absence of any commercial or financial relationships that could be construed as a potential conflict of interest.
